# Isolation and Identification of Cytotoxic Compounds Present in Biomaterial Life^®^

**DOI:** 10.3390/ma15030871

**Published:** 2022-01-24

**Authors:** Maria Beatriz Ferreira, Nelson A. M. Pereira, Carlos Miguel Marto, Miguel Cardoso, Inês Amaro, Ana Coelho, José Saraiva, Gianrico Spagnuolo, Manuel Marques Ferreira, Marta Piñeiro, Teresa M. V. D. Pinho e Melo, Maria Filomena Botelho, Eunice Carrilho, Anabela Paula, Mafalda Laranjo

**Affiliations:** 1 Institute of Integrated Clinical Practice, Faculty of Medicine, University of Coimbra, 3000-354 Coimbra, Portugal; mariabeatriz.ferreira@hotmail.com (M.B.F.); miguel.cardoso16@gmail.com (M.C.); ines.amaros@hotmail.com (I.A.); anasofiacoelho@gmail.com (A.C.); ze-93@hotmail.com (J.S.); eunicecarrilho@gmail.com (E.C.); 2 Coimbra Chemistry Centre (CQC)-Institute of Molecular Sciences (IMS) and Department of Chemistry, University of Coimbra, 3004-535 Coimbra, Portugal; npereira@qui.uc.pt (N.A.M.P.); mpineiro@qui.uc.pt (M.P.); tmelo@ci.uc.pt (T.M.V.D.P.e.M.); 3 Institute of Experimental Pathology, Faculty of Medicine, University of Coimbra, 3000-354 Coimbra, Portugal; cmiguel.marto@uc.pt; 4 Institute of Biophysics, Faculty of Medicine, University of Coimbra, 3000-354 Coimbra, Portugal; mfbotelho@fmed.uc.pt; 5 Coimbra Institute for Clinical and Biomedical Research (iCBR), Area of Environment, Genetics and Oncobiology (CIMAGO), Faculty of Medicine, University of Coimbra, 3000-354 Coimbra, Portugal; m.mferreira@netcabo.pt; 6 Center for Innovative Biomedicine and Biotechnology (CIBB), University of Coimbra, 3000-548 Coimbra, Portugal; 7 Clinical Academic Center of Coimbra (CACC), 3000-354 Coimbra, Portugal; 8 Department of Neurosciences, Reproductive and Odontostomatological Sciences, University of Naples “Federico II”, 80125 Napoli, Italy; gspagnuo@unina.it; 9 Institute of Dentistry, I. M. Sechenov First Moscow State Medical University, 119146 Moscow, Russia; 10 Institute of Endodontics, Faculty of Medicine, University of Coimbra, 3000-354 Coimbra, Portugal

**Keywords:** direct pulp capping, biomaterial, calcium hydroxide, cytotoxicity, odontoblasts

## Abstract

Direct pulp capping consists of a procedure in which a material is directly placed over the exposed pulp to maintain dental vitality. Although still widely used in clinical practice, previous in vitro studies found that the biomaterial Life^®^ presented high cytotoxicity, leading to cell death. This study aimed to identify the Life^®^ constituents responsible for its cytotoxic effects on odontoblast-like cells (MDPC-23). Aqueous medium conditioned with Life^®^ was subjected to liquid–liquid extraction with ethyl acetate. After solvent removal, cells were treated with residues isolated from the organic and aqueous fractions. MTT and Trypan blue assays were carried out to evaluate the metabolic activity and cell death. The organic phase residue promoted a significant decrease in metabolic activity and increased cell death. On the contrary, no cytotoxic effects were observed with the mixture from the aqueous fraction. Spectroscopic and spectrometric methods allowed the identification of the toxic compounds. A mixture of the regioisomers *ortho*, *para,* and *meta* of *N*-ethyl-toluenesulfonamide was identified as the agent responsible for the toxicity of biomaterial Life^®^ in MDPC-23 cells. These findings contribute to improving biomaterial research and development.

## 1. Introduction

Advanced dental caries, iatrogenic events, and severe trauma are causes of pulp tissue exposure. Over time, different strategies have been developed to treat exposed pulp, including direct and indirect pulp capping and pulpotomy [1]. These treatments aim to preserve the pulp tissue and stimulate the repair of the exposed area through the production of a mineralized tissue barrier [2]. Direct pulp capping consists of a procedure in which a biomaterial is placed directly over the exposed pulp to maintain pulp vitality [3]. This could be considered as a minimally invasive approach in the treatment of deep caries and, if successful, can avoid the necessity to perform a root canal treatment [4,5].

Materials with an active ingredient based on calcium hydroxide (Ca(OH)_2_) are among the most popular therapeutic agents used in direct pulp capping. These materials’ ability to release hydroxyl ions (OH^-^) and calcium ions (Ca^2+^) promotes pulp cells’ differentiation into odontoblast-like cells and, consequently, dentin barrier formation in the exposed zone [6,7]. For decades, calcium hydroxide has been considered the gold-standard biomaterial for pulp capping since it has excellent antimicrobial properties and can induce pulp repair mechanisms. However, the formation of this reparative dentin may not be due to its bioinductive capacity but the result of a defense mechanism of pulp tissue against a natural irritant such as calcium hydroxide [8]. The high pH (12.5) causes the liquefaction necrosis of the pulp surface, leading to the formation of a necrotic layer at the material–dental pulp interface [8]. This layer can reach 1.5 mm in depth, compromising the vascularization of this area and interfering with coagulation [9]. This caustic action of Ca(OH)_2_ reduces the underlying pulp size to 0.7 mm [10].

Many studies also indicate a decline in the rate of pulp vitality over time when Life^®^ or other calcium hydroxide materials are used [8,11,12]. Numerous factors may contribute to the low success rates observed [9]. The fact that it produces a faulty dentinal bridge, its gradual dissolution, and the inability to promote a proper long-term seal raise some concerns when using this pulp capping biomaterial [6]. The development of new compounds such as mineral trioxide aggregates allowed better results due to decreased necrosis and inflammation levels and more stable dental bridges [2,3]. However, these materials are associated with tooth structure discolorations, complex manipulation, and prolonged settings [13]. Recently, other alternatives have been developed, such as tricalcium silicate-based cements [14,15].

In a previous study by Paula et al. [16], different materials used in direct pulp capping were evaluated. The authors concluded that the biomaterial Life^®^ (Kerr Hawe, Bioggio, Switzerland), a cement based on calcium hydroxide, caused a decrease in metabolic activity and increased cell death. A two-paste system forms this biomaterial: the base paste, composed of zinc oxide (ZnO), calcium hydroxide (Ca(OH)_2_), and calcium oxide (CaO), and the catalyst paste, constituted of methyl salicylate (C_8_H_8_O_3_), barium sulfate (BaSO_4_), titanium dioxide (TiO_2_), and 2,2-dimethylpropane-1,3-diol (C_5_H_12_O_2_). The mixture of these two pastes triggers chemical reactions that lead to calcium disalicylate complexes, which form the basis of this cement [17,18]. As the biomaterial Life^®^ presents some interesting properties and is still widely used in clinical practice [19], it is very relevant to understand the causes of its diminished biocompatibility. We hypothesize that there is a component, other than calcium salts, that is responsible for Life^®^’s cytotoxicity. Therefore, this work aimed to identify the chemical component(s) potentially responsible for its cytotoxicity.

## 2. Materials and Methods

### 2.1. Biomaterial

Biomaterial Life^®^ was used in this study (LOT 6775374 and LOT 6880919; Kerr Hawe, Bioggio, Switzerland). Conditioned media were obtained as previously described [16]. Briefly, the paste system was mixed in equal parts, manipulated, and placed in polyvinyl chloride molds at 37 °C for 24 h. The resulting pellets were sterilized and incubated in a cell culture medium composed of Dulbecco’s Modified Eagle’s Medium (DMEM; Sigma D-5648, Sigma-Aldrich Corporation, St. Louis, MO, USA) enriched with 10% fetal bovine serum (FBS; Sigma F7524; Sigma-Aldrich Corporation, St. Louis, MO, USA), for 24 h at 37 °C. A surface area of 250 mm^2^ per milliliter was used. This was considered the 100% concentration, and sequential dilutions of conditioned media were prepared with fresh cell culture media [20]. For simplification, these media will be named dCM.

Given the complexity of the cell culture medium composition, conditioned water was prepared in parallel. For this, autoclaved ultrapure water was incubated with the same biomaterial contact area and extraction conditions. The use of conditioned water solutions simplifies the liquid–liquid extraction, so, for the chemistry studies, conditioned water solutions were used.

Besides testing dCM, conditioned water was diluted in an equal volume of double-concentrated DMEM to obtain a 50% conditioned medium for the biological studies. Sequential dilutions were further prepared with fresh cell culture media. For simplification, these media will be named wCM.

### 2.2. Liquid–Liquid Extraction

After preparing the water conditioned with the biomaterial Life^®^, as previously described, the solution (20 mL) was subjected to extraction with ethyl acetate (2 × 20 mL). The combined organic layers were dried over anhydrous sodium sulfate, and the solvent was evaporated under reduced pressure.

For the biological studies, the aqueous solution was later solubilized in cell culture media (DMEM, 10% FBS), and the organic fraction was solubilized in dimethyl sulfoxide (DMSO; Sigma-Aldrich Corporation, St. Louis, MO, USA).

### 2.3. Column Chromatography Separation

The compounds from the organic residue were isolated by flash column chromatography, using silica gel 60 as the stationary phase (0.035–0.070 mm; Acros Organics, Thermo Fisher Scientific, Waltham, MA, USA and dichloromethane (Sigma-Aldrich Corporation, St. Louis, MO, USA) as an eluent. Elution controls were performed by thin-layer chromatography (TLC), using pre-coated silica gel plates, namely Macherey-Nagel Xtra SIL G/UV254 (Macherey-Nagel, Dueren, Germany).

### 2.4. Chemical Analysis

Proton and carbon nuclear magnetic resonance spectra, ^1^H NMR and ^13^C NMR, were recorded using Bruker Avance III spectrometers (Bruker Corporation, Billerica, MA, USA), operating at 400 MHz (^1^H NMR) and 100 MHz (^13^C NMR). Deuterated chloroform (CDCl_3_) was used as the solvent. The values of the chemical shifts (*δ*) are presented in parts per million (ppm) in relation to the internal standard tetramethylsilane (TMS), and the values of the coupling constants (*J*) are expressed in Hz. Analysis by gas chromatography coupled with mass spectrometry (GC-MS) was performed on an Agilent Technologies 7820 Series GC System gas chromatograph coupled to an Agilent Technologies 5975 MSD System mass spectrometer (Agilent Technologies, Santa Clara, CA, USA).

### 2.5. Cell Cultures

In vitro cellular procedures followed the ISO 10993-5 (“International Standard of Biological Evaluation of Medical Devices—part 5: tests for in vitro cytotoxicity”) [21]. The mouse odontoblast MDPC-23 cell line was used as a model to evaluate the biocompatibility of dental materials [8] and was provided by Professor Jacques Nör (University of Michigan, Ann Arbor, MI, USA). Cells were cultured in DMEM enriched with 10% FBS, 250 μM sodium pyruvate (Gibco 11360, Thermo Fisher Scientific, Waltham, MA, USA), and 1% antibiotics (100 U/mL of penicillin and 10 μg/mL streptomycin; Sigma A5955, Sigma-Aldrich Corporation, St. Louis, MO, USA). Cell suspensions with 50,000 cells/mL were prepared and distributed by multiwell plates for every assay. Plates (12 or 96 wells) were incubated overnight to allow cell attachment.

### 2.6. Cytotoxicity Studies

Life^®^ was administered to the cell cultures at a concentration of 50% using both dCM and wCM. Extracts of the organic and aqueous fractions, obtained from liquid–liquid extraction, were administered in concentrations of 6.25%, 12.5%, 25%, 50%, and 100%. Control cultures not subjected to any treatment were included in every plate. A second control was included in the organic extract assays, corresponding to 1% DMSO, the vehicle of solubilization of this phase. Cell cultures were incubated for 24, 72, and 120 h.

### 2.7. Metabolic Activity

To evaluate the effect of biomaterials on cell metabolic activity, the MTT assay (3-(4,5-dimethylthiazol-2-yl)-2,5-diphenyltetrazolium bromide) was performed. First, the cultures were washed with phosphate-buffered saline (PBS), constituted of 137 mM sodium chloride (NaCl, Sigma S7653, Sigma-Aldrich Corporation, St. Louis, MO, USA), 2.7 mM potassium chloride (KCl, Sigma P9333, Sigma-Aldrich Corporation, St. Louis, MO, USA), 10 mM sodium hydrogen phosphate (NaH_2_PO_4_, Sigma S5011, Sigma-Aldrich Corporation, St. Louis, MO, USA), and 1.8 mM potassium hydrogen phosphate (KH_2_PO_4_, Sigma P0662, Sigma-Aldrich Corporation, St. Louis, MO, USA), pH 7.4. Subsequently, the cultures were incubated with the MTT solution (0.5 mg/mL; Sigma M2128, Sigma-Aldrich Corporation, St. Louis, MO, USA), prepared in PBS at 37 °C overnight. To solubilize the formazan crystals, equal volumes of 0.04 M hydrochloric acid solution in isopropanol were added to each well. The plates were left under stirring for 30 min. Absorbance was read on the EnSpire^®^ spectrophotometer (PerkinElmer Inc., Shelton, CT, USA) at a wavelength of 570 nm, with the absorbance of 620 nm as a reference value. Metabolic activity was expressed as a percentage of the metabolic activity of cultures subjected to conditioned media in relation to the metabolic activity of control cell cultures.

### 2.8. Cell Viability

The Trypan blue exclusion test was performed. Samples of each cell suspension were dyed with equal amounts of Trypan blue solution (Sigma T8154, Sigma-Aldrich Corporation, St. Louis, MO, USA). Stained (non-viable) and unstained (viable) cells were counted in a Neubauer chamber under the microscope (Nikon Eclipse TS 100, Tokyo, Japan).

### 2.9. Statistical Analysis

The statistical analysis was performed using the Prism 9 software. The normal distribution of quantitative variables was accessed with the Shapiro–Wilk test. Metabolic activity was compared with normalization values, using the t-test where a normal distribution and homogeneity of variances were verified, and the Wilcoxon test otherwise. As applicable, the mean comparisons were performed with the one-sample t-test or Mann–Whitney. Cell viability results were compared using one-way ANOVA after confirming the criteria of normality and homogeneity of variances. Multiple comparisons were made between the groups with the respective Bonferroni correction. A significance value of 5% was considered.

## 3. Results

### 3.1. Metabolic Activity

**Figure 1:**
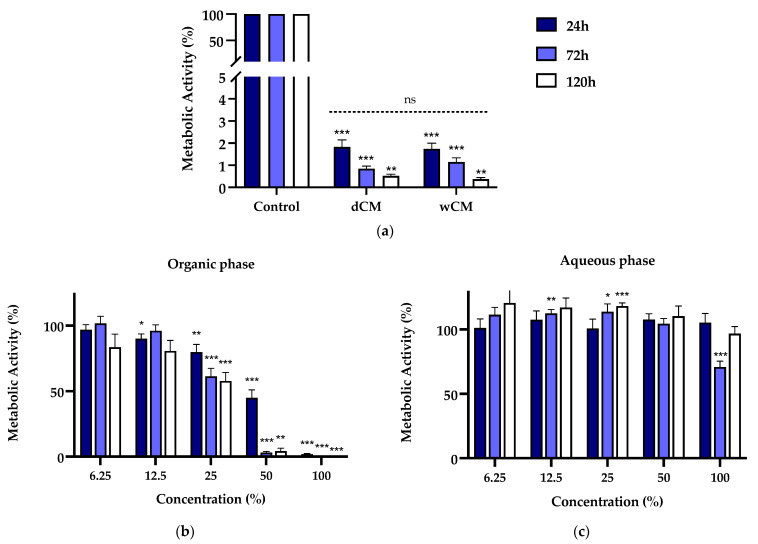
(**a**) Metabolic activity of MDPC-23 cells exposed for 24, 72, and 120 h to Life^®^ dCM and wCM at a 50% concentration. For each incubation time, non-significant differences were found between dCM and wCM. (**b**) Metabolic activity of MDPC-23 cells exposed for 24, 72, and 120 h to the organic phase extract of the Life^®^ wCM. (**c**) Metabolic activity of MDPC-23 cells exposed for 24, 72, and 120 h to the aqueous phase extract of the Life^®^ wCM. The results represent the mean and standard error of *n* ≥ 8. Statistically significant differences regarding the control are represented by *, where * means *p* < 0.05, ** *p* < 0.01, *** *p* < 0.001, and ns means not significant.

The metabolic activity of the MDPC-23 cells incubated with Life^®^-conditioned medium and organic and aqueous phases is represented in Figure 1. The treatment with Life^®^-conditioned medium led to an extreme metabolic activity decrease to values below 2% (*p* < 0.004) for the incubation periods of 24, 72, and 120 h (Figure 1a). This was observed both by using dCM, as we did in our previous investigation [17], and wCM. Moreover, we verified no significant differences in the metabolic activity of dCM- versus wCM-treated cell cultures, irrespective of the incubation time studied.

A differential cell response was observed when cell cultures were subjected to wCM organic and aqueous phases. The organic extract led to a concentration- and incubation time-dependent loss of metabolic activity (Figure 1b). In particular, for concentrations equal or superior to 25%, there was a significant decrease in metabolic activity to 79.88 ± 5.75% (*p* = 0.0049), 61.40 ± 6.12% (*p* = 0.0005), and 57.82 ± 6.52%(*p* < 0.0001) for the respective incubation periods of 24, 72, and 120 h. The organic extract at a concentration of 50% determined a significant decrease in the metabolic activity to 44.99 ± 6.07% (*p* < 0.0001), 3.21 ± 0.78% (*p* < 0.0001), and 4.35 ± 2.11 (*p* = 0.0078). At 100% concentration, metabolic activity below 2% (*p* ≤ 0.0005) was obtained.

Despite the organic phase’s substantial effects, overall, the treatment with the aqueous phase extract did not determine substantial changes in metabolic activity compared to untreated control cell cultures (Figure 1c). Aside from a significant decrease to 70.90 ± 6.576 (*p* = 0.007) at 72 h, which was not corroborated by the longer incubation time, no other metabolic activity losses were seen. Statistically significant increases were found for 12.5% and 25% concentrations, albeit biologically negligible.

### 3.2. Cell Viability

**Figure 2:**
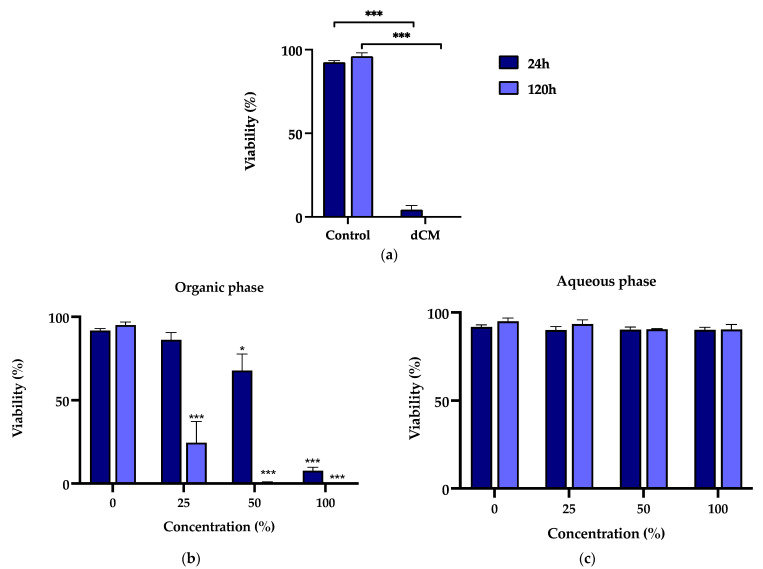
(**a**) Viability of MDPC-23 cells exposed for 24 and 120 h to Life^®^ dCM at a 50% concentration (**b**) Viability of MDPC-23 cells exposed for 24 and 120 h to the organic phase extract of the Life^®^ wCM. (**c**) Viability of MDPC-23 cells exposed for 24, 72, and 120 h to the aqueous phase extract of the Life^®^ wCM. The results represent the mean and standard deviation of *n* = 3. Statistically significant differences regarding the control are represented by *, where * means *p* < 0.05, and *** *p* < 0.001.

In a complementary manner to evaluating metabolic activity, the Trypan blue exclusion test was performed to assess cell viability. The cell viability of the MDPC-23 cells incubated with Life^®^-conditioned media and the organic and aqueous phases is represented in Figure 2. Life^®^-conditioned media radically impaired cell viability (Figure 2a). For a 24 h exposure period to Life^®^-conditioned media, at a 50% concentration, reduced cell viability of 4.27 ± 2.52% (*p* < 0.0001) was found. Viability was almost absent for a 120 h exposure period (*p* < 0.0001).

Corroborating the previous findings, the viability of MDPC-23 cells subjected to the Life^®^ wCM organic phase was decreased in a concentration- and time-dependent manner (Figure 2b). In this case, comparisons were made between the groups subjected to the organic fraction and the DMSO control group, the solvent used to solubilize the organic fraction. For an incubation period of 24 h, cells exposed to a concentration of 50% and 100% showed a loss of cell viability to 67.82 ± 9.88% (*p* = 0.0148) and 7.44 ± 2.03% (*p* < 0.0001), respectively. After 120 h of exposure, for the 25% concentration, cell viability of 24.59 ± 12.68 (*p* < 0.001) was found, while, for the higher concentrations, it was negligible (*p* < 0.0001). Notwithstanding, MDPC-23 cells subjected to the aqueous phase of Life^®^ wCM remained with values above 90% for all concentrations and time exposures (Figure 2c).

### 3.3. Chemical Analysis of the Organic Extract

The water conditioned with Life^®^ was subjected to liquid–liquid extraction with an organic solvent. Then, the crude mixture obtained from the organic extract was purified by flash column chromatography. The isolated products were identified using different spectroscopic techniques.

**Figure 3:**
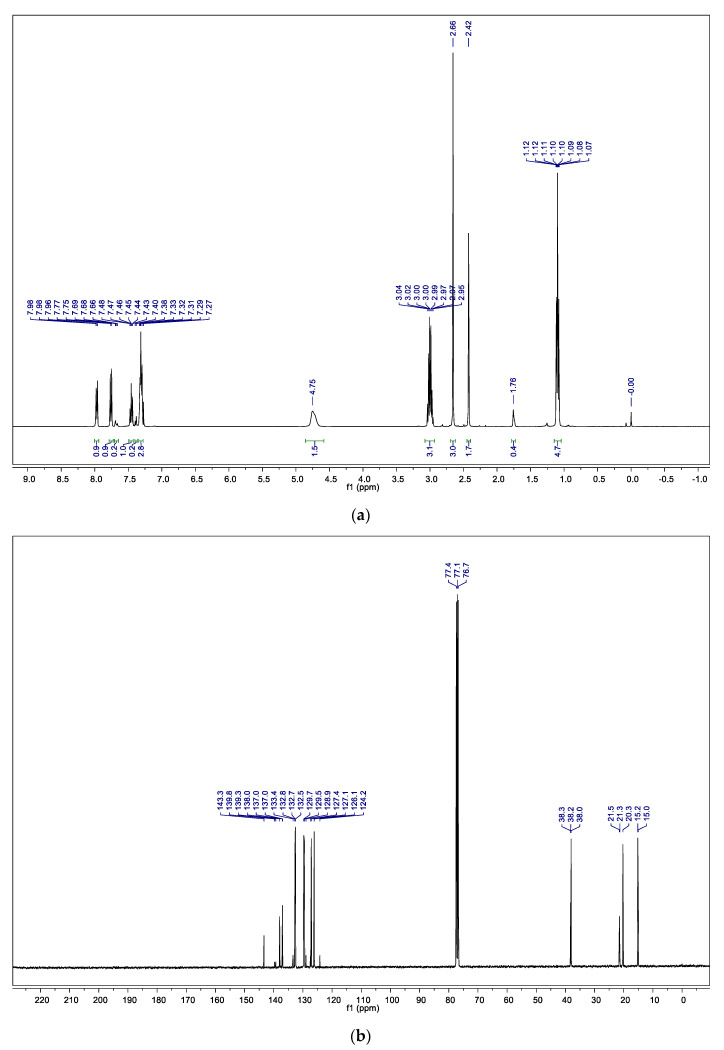
(**a**) ^1^H NMR spectrum of the organic extract in CDCl3; (**b**) ^13^C NMR spectrum of the organic extract in CDCl3.

The first analysis was carried out by nuclear magnetic resonance spectroscopy, ^1^H NMR and ^13^C NMR. Considering the composition of Life^®^ (described by the manufacturer) and the chemical shifts observed in the aromatic region of ^1^H NMR spectrum (7–8 ppm, Figure 3a), the presence of methyl salicylate or derivatives was evaluated. However, the presence of multiplets in the typical region of sp3 carbons at the ^1^H NMR (0–5 ppm) is indicative of the incorporation of a chain with at least two saturated carbons. Additionally, the lack of peaks in the ^13^C NMR spectrum (Figure 3b) between 160 and 200 ppm, typically assigned to carbons of carbonyl groups, led us to exclude this possibility.

**Figure 4:**
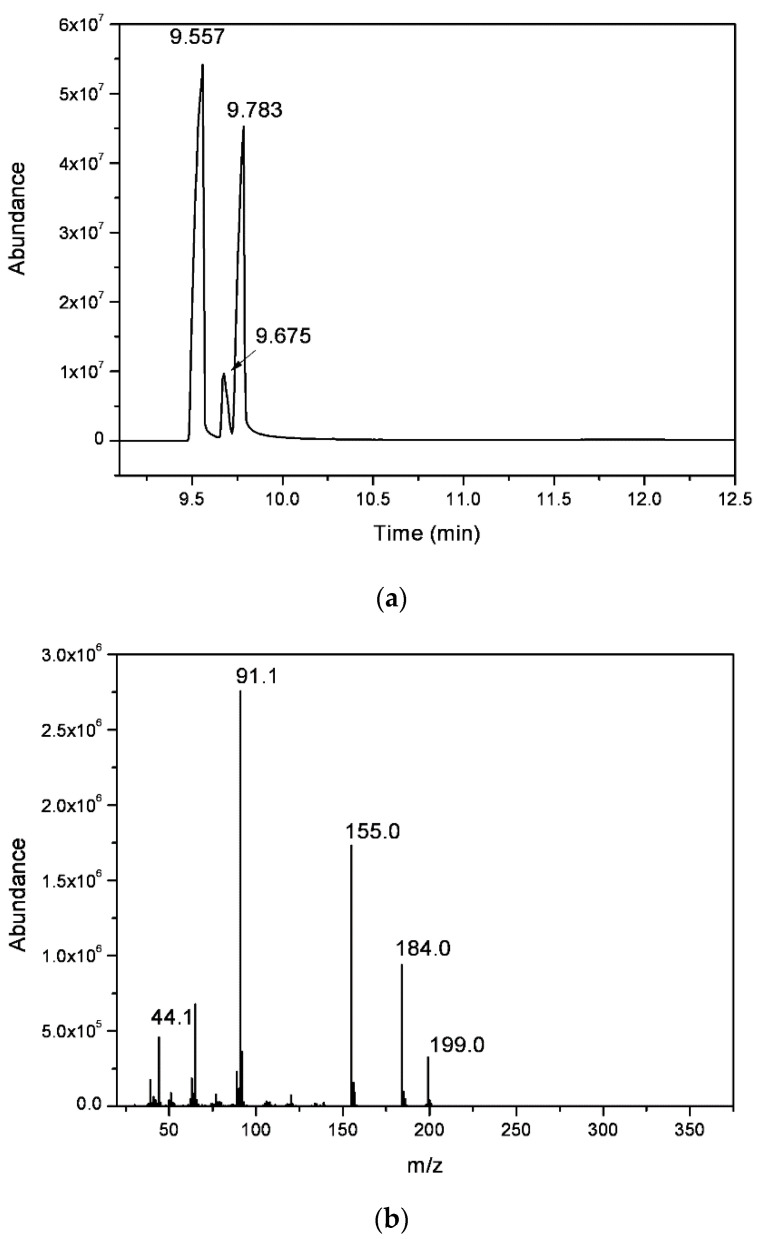
(**a**) GC chromatogram and (**b**) electron impact ionization mass spectrum (GC peak with r.t. = 9.68 min) of the organic extract.

**Figure 5:**
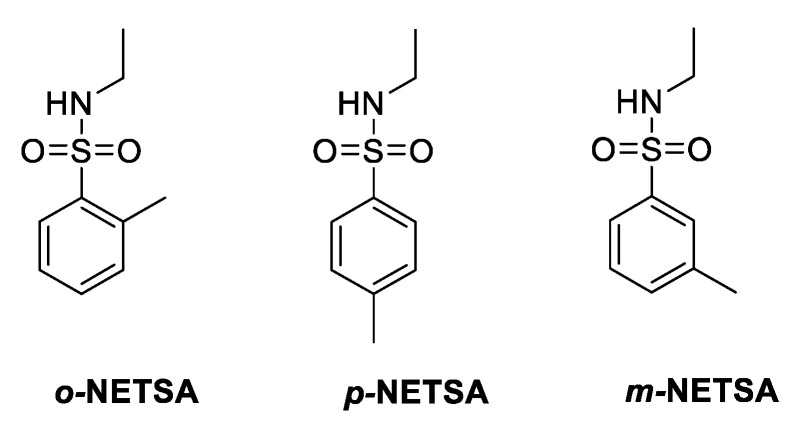
Chemical structures of the regioisomers, *ortho*, *para*, and *meta*, of N-ethyl-toluenesulfonamide (NETSA), compounds present in the organic extract.

The chromatogram obtained from the gas chromatography–mass spectrometry analysis (Figure 4a) revealed the presence of three compounds, with close elution retention times: 9.56, 9.68, and 9.78 min. All the peaks presented similar mass spectra, with a molecular ion peak at *m/z* = 199, showing a similar fragmentation pattern, with a higher value than expected for the methyl salicylate derivative (molecular weight = 152 g mol^−1^). Additionally, fragmentation peaks at *ca m/z* = 91 are typical of compounds with a toluene moiety, and *m/z* = 44.1 could be assigned to the ethyl amine fragment (Figure 4b). The data suggest that the organic residue isolated contained a mixture of three regioisomers, *ortho* (57%) and *para* (36%) and traces of the *meta* (7%) *N*-ethyl-toluenesulfonamide (NETSA, Figure 5). In this context, the organic extract is constituted by a mixture of the two major isomers

## 4. Discussion

A biomaterial used for direct pulp capping must adhere to the dental surface and guarantee correct sealing. Ideally, it should also not be resorbable, toxic, carcinogenic, or genotoxic, and it should present biocompatibility and bioactivity [1]. Since the biomaterial Life^®^ has shown high cytotoxicity compared to other biomaterials [16], it encouraged the development of this work to identify the component(s) responsible for the observed cell death.

Therefore, the results from a previous study were replicated regarding the Life^®^-conditioned medium [16]. However, as cell culture media are complex solutions, identifying single components by chemistry techniques would be challenging. Thus, we investigated whether the cellular response obtained for Life^®^-conditioned media (dCM) would be the same as that obtained for cell culture media prepared in previously conditioned water solutions (wCM). The latter would make it feasible for subsequent chemistry studies. MTT results show that, for exposure to Life^®^ dCM and wCM at a concentration of 50%, the metabolic activity was shallow, without significant differences between the two conditions. Besides confirming the cytotoxic effect of the biomaterial in the cell culture MDPC-23, these results also guaranteed the viability of the methodology adopted in this study.

The MTT assay determines metabolic activity and is widely used to assess biomaterial cytotoxicity [22,23]. However, this analysis alone becomes insufficient since the decrease in metabolic activity does not always necessarily result from cell death. Therefore, the Trypan blue exclusion test was carried out to assess cell viability. Cells exposed to Life^®^ dCM at a concentration of 50% showed viability to values close to zero. Thus, the results from this test allowed us to guarantee that the decrease in metabolic activity would be associated with cell death. According to the ISO10993-5, a biomaterial is considered cytotoxic when the viability decreases for more than 30% [21]. Importantly, the use of standardized and reproducible methodologies increases the confidence in the obtained results and contributes to decreasing the gap between in vitro models and clinical application [24].

It is important to note that if a material is toxic in vitro, it may not necessarily reveal high cytotoxicity in vivo. However, there is scientific clinical evidence that calcium hydroxide-based materials are associated with lower success rates when compared with other biomaterials used in the direct pulp capping technique [1,14,25,26]. Therefore, liquid–liquid separation was carried out to identify the substances present in the biomaterial responsible for the already known cytotoxicity. This separation was made between conditioned ultrapure water (wCM) and ethyl acetate to extract the organic components from the aqueous medium. The extracts from the two phases were evaluated in MDPC-23 cells as before, and MTT and Trypan blue assays were performed. Interestingly, no cytotoxicity was observed within the aqueous phase extract. Conversely, it was proven that organic constituents were responsible for the cytotoxicity since there was a significant decrease in metabolic activity and cell viability for concentrations equal to or greater than 25%.

Various spectroscopic techniques were used to identify the constituents of the organic extract. ^1^H NMR, ^13^C NMR, and GC-MS spectra showed that the organic extract is a mixture of three regioisomers, *ortho*, *para*, and *meta* of N-ethyl-toluenesulfonamide (NETSA). In fact, after a more in-depth search of the Life^®^ composition, we confirmed the presence of NETSA. At the time, this constituent was not mentioned in the European safety data sheet but was referred to in the American safety data sheet. The manufacturer recently corrected this situation [27].

The experimental spectra data (Figure 3 and Figure 4) are in good agreement with those described in the literature for *p*-NETSA [28]. The percentage of each isomer was obtained from the relative area of the corresponding peak of the GC chromatogram, supported by the assignment established by the ^1^H NMR spectrum. For example, the signals of the four protons of the aromatic moiety appearing as two doublets at 7.97 and 7.76 ppm, and the protons of the aromatic substituted CH_3_ group as a singlet, with a chemical shift at 2.42 ppm, match very well with the data reported for the *para* isomer [28]. In this context, the organic extract is constituted by a mixture of the two major isomers *ortho* and *para* and traces of the *meta* derivative.

NETSA is used as a plasticizer in resins, cellulose acetate, nitrocellulose, lacquers, adhesives, and paints; consequently, it has been confirmed as a contaminant in water and food products [29]. Despite the scarce data on the toxicological properties of these compounds, some mutagenic effect has been reported [30] and, therefore, its use should be a matter of concern. Although new materials have improved properties, calcium hydroxide cements still present interesting properties, namely excellent antimicrobial action and a moderate success rate. The discovery of this material component responsible for the cytotoxic effect can support the notion that, in the future, other calcium hydroxide-based materials can be developed for use in dentistry. Our data show that the replacement of NETSA with other natural-based plasticizers should be considered, with improved properties and without negative consequences to human health [31].

This study also highlights the utility of chemical techniques for developing and evaluating biomaterials, which have often a complex constitution with dozens of components [32,33,34]. Additionally, chemical reactions occur during their manipulation, application, and later when in contact with the living tissues [19,35,36]. This can lead to the emergence of different chemical agents, which need to be identified and evaluated to ensure safety.

## 5. Conclusions

This study confirmed Life^®^’s toxicity in MDPC-23 cells, validating previous studies and identifying *N*-ethyl-*o/p/m*-toluenesulfonamides as responsible for its cytotoxicity. These results also prove the need for greater accuracy regarding the description of dental biomaterials, especially when it comes to residual compounds that may influence the product’s biocompatibility. Although biomaterials with improved characteristics are already commercially available, Life^®^ continues to be widely used in clinical practice. However, considering our results, and since materials with improved characteristics and higher clinical success rates are available, its use is not recommended.

## Data Availability

The data presented in this study are available in the manuscript.
